# Amplitude concentration in a phase-modulated spectrum due to femtosecond filamentation

**DOI:** 10.1038/srep43367

**Published:** 2017-03-07

**Authors:** J. V. Thompson, P. A. Zhokhov, M. M. Springer, A. J. Traverso, V. V. Yakovlev, A. M. Zheltikov, A. V. Sokolov, M. O. Scully

**Affiliations:** 1 Texas A&M University, College Station, TX, 77843, USA; 2 M.V. Lomonosov Moscow State University, Physics Department, International Laser Center, 119992 Moscow, Russia; 3 Baylor University, Waco, TX, 76798, USA

## Abstract

We present a method by which the spectral intensity of an ultrafast laser pulse can be accumulated at selected frequencies by a controllable amount. Using a 4-f pulse shaper we modulate the phase of the frequency components of a femtosecond laser. By inducing femtosecond filamentation with the modulated pulse, we can concentrate the spectral amplitude of the pulse at various frequencies. The phase mask applied by the pulse shaper determines the frequencies for which accumulation occurs, as well as the intensity of the spectral concentration. This technique provides a way to obtain pulses with adjustable amplitude using only phase modulation and the nonlinear response of a medium. This provides a means whereby information which is encoded into spectral phase jumps may be decoded into measurable spectral intensity spikes.

The production of femtosecond laser pulses has played a significant role in the advancement of modern science. In addition to their ultrashort pulse duration, femtosecond laser pulses offer a broad spectral bandwidth and a high instantaneous intensity for each pulse^1^. Because of these properties, femtosecond pulses have become a very attractive tool with a wide variety of applications including filamentation^2^,^3^, communication^4^,^5^, coherent control^6^,^7^, metrology^8^, micromachining^9^, and spectroscopy^10^,^11^,^12^. Of particular interest here is the fact that femtosecond filaments^13^ are extremely versatile in application, and have been formed in air^14^, water^15^,^16^, and other condensed media^3^,^17^. They are used in various types of spectroscopy including remote sensing of the atmosphere^18^,^19^ and filament induced breakdown spectroscopy^20^. They have been used for sub-diffraction imaging^21^, and as a trigger and guide to high-voltage discharges^22^. The spectral broadening and pulse compression characteristics of femtosecond filamentation have also been useful in creating pulses with few-cycle pulse duration^23^,^24^,^25^. The filamentation of femtosecond pulses is accompanied by a number of physical effects including conical emission^26^, white-light continuum generation, ionization, and nonlinear absorption^27^,^28^. In addition to this, femtosecond filaments can propagate for long distances^18^,^29^, making them an attractive mode for laser/filament based communications^5^.

In this article, we investigate an approach using phase only pulse shaping with femtosecond filamentation to convert energy from one part of the spectrum of an ultrafast laser pulse to another part of the spectrum. This method may also be used as a possible way to communicate information. In particular, we demonstrate experimental and numerical results wherein the spectral amplitude of a femtosecond laser pulse is accumulated by a controllable amount at selected frequencies. These results have direct impact on the use of femtosecond lasers and filamentation in communication schemes, since in this method, information can be encoded onto a laser pulse with a phase mask, and decoded again as measurable intensity spikes.

A direct result of the broad bandwidth associated with femtosecond pulses is the ability to shape the temporal profile by modulation of the spectral components. This temporal shaping can be done using a 4-f pulse shaper, which consists of a diffraction grating to spatially disperse the pulse into separate frequency components, a lens to perform a spatial Fourier transform, a spatial phase or amplitude mask to modulate the shape, and another lens and grating to recombine and collimate the pulse^30^. Various pulse shaper designs utilize modulation of the spectral amplitude, spectral phase, or both to temporally shape ultrafast pulses. We use a computer controlled liquid crystal spatial light modulator to apply a phase mask to the pulse as it propagates through the 4-f pulse shaper^31^,^32^.

One technique that can be implemented with shaped femtosecond pulses is spectral hole filling, which was developed by Warren *et al*. as a way to detect the onset of two photon absorption^33^,^34^. Because nonlinear processes such as two-photon absorption and self-phase modulation alter the frequency content of a pulse, if the input laser pulse contains a spectral hole (i.e. region of missing spectral components), the effect of these nonlinear processes will fill in some of the missing spectral components. Thus, by monitoring the hole in the spectral domain, it is possible to detect the onset of the nonlinear process. This has been shown for both two photon absorption^33^ and self-phase modulation^35^. Spectral hole filling has been demonstrated not only to refill a hole but to overfill it via self-phase modulation^36^. This overfilling effect is explained by examining the Fourier decomposition of the pulse into a set of modes in the time domain. A spectral hole is modeled as the destructive interference between frequency modes of the original pulse and quasi-continuous waves of the same frequency. As the pulse undergoes self-phase modulation, the phase of the modes of the original pulse are shifted, while the phase of the quasi-continuous waves are not. Thus, the destructive interference becomes less destructive or even constructive interference depending on the phase shift caused by self-phase modulation. This overfilling was shown to be an intensity dependent effect. Similar studies have investigated spectral hole filling in supercontinuum generation in optical fibers due to phase modulation^37^.

In this paper, we present a technique similar to spectral hole overfilling in the regime of femtosecond filamentation. Here, the effect is driven by a phase shift applied by a pulse shaper to a narrow band of frequencies in the pulse, rather than by a spectral hole. While femtosecond filamentation is accompanied by the generation of new frequencies, we focus here on the role and importance of intensity dependent attenuation in the process of amplitude concentration. This is because of the key role that nonlinear absorption plays in filamentation in water^38^. It is important to note that any other intensity dependent attenuation method (other than filamentation) should yield similar results. The critical point is that ultrafast pulses with high intensity (above threshold) are attenuated by filamentation, whereas pulses with low intensity (below threshold) do not undergo filamentation. This effect is dependent upon the phase shift applied to the frequency components of the pulse. Thus, a pulse shaper provides control over which frequencies to enhance and by how much to enhance them (by changing the phase shift).

## Theoretical Description

A laser pulse can be completely described by its Fourier decomposition *A(ω*), where


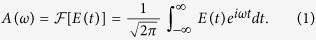


Here *E(t*) is the electric field, *ω* is the angular frequency, and 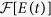 represents the Fourier transform of *E(t*). In this experiment, a pulse shaper performs a spectral transformation


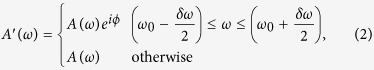


where *δω* is the width of a narrow spectral window about the center frequency *ω*_0_. Note that if the applied phase shift is *ϕ* = *π*, the multiplicative factor becomes −1, denoting a phase flip. After this transformation, the pulse can be written in terms of a superposition


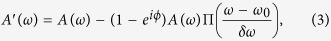


where Π is the unit box function. Note that in the case that the applied phase shift is replaced by a spectral hole (i.e. replace *e*^*iϕ*^ with 0), this equation is equivalent to the explanation for spectral hole overfilling discussed previously^36^. The modified pulse is again treated as a superposition of two waves; however, here the amplitude of the second wave is dependent upon the phase shift applied.

The Fourier transform obeys the principle of superposition, 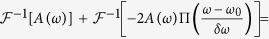 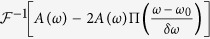. Heuristically the first term represents the broad-bandwidth pulse before transformation by the spectrometer. The second term is a narrow-bandwidth pulse superposed on the original broad-bandwidth pulse. The amplitude of this narrow-bandwidth pulse in the spectral domain varies little over the region within the narrow unit box, and may be approximated as a constant. Thus this term under inverse Fourier transform becomes effectively


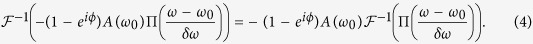


**Figure 1:**
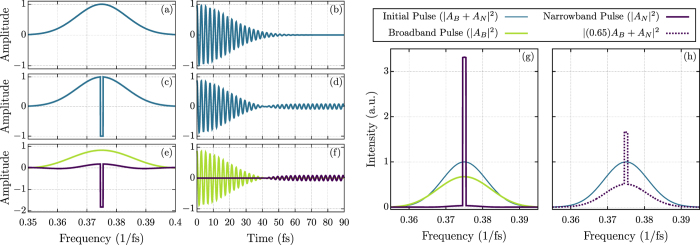
Overview of theory in frequency domain (first column) and time domain (second column). A narrow region of frequencies in a laser pulse (**a**,**b**) undergoes a *π* phase shift (**c**,**d**). The amplitude of the shaped pulse is described as the sum of two pulses (**e**,**f**). We assume a symmetric pulse in the time domain, however, for better clarity, we only show half of the pulse in the figure. The spectra of these pulses are shown in (**g**,**h**). The original pulse is given for reference (blue). The spectrum of the separate components (**g**, violet, green) of the shaped pulse are obtained if the other component is removed completely. The scenario of slight attenuation of the broadband pulse is also shown ((**h)** violet, dashed).

The inverse Fourier transform of the unit box is 
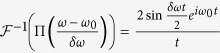
. As such, the narrow-bandwidth slice of the modulated pulse in the frequency domain is effectively approximated in the time domain with an envelope whose temporal width is inversely proportional to the modulated bandwidth *δω*. Because the modulated bandwidth is spectrally narrower than the original pulse, the time-domain representation of the modulated bandwidth is a much longer pulse. Thus, the superposition principle allows the modulated pulse in the time domain to be described as the original time-domain pulse overlapped with a low-amplitude much longer pedestal pulse. These results are depicted in Fig. 1.

At this point, the Fourier description of the modulated pulse in the time domain as the superposition of a short pulse and a long pulse has been conducted entirely in the linear regime. In the nonlinear regime, the intensity of the pulse is a relevant factor in its propagation; in particular, the attenuation of a sufficiently intense pulse can be intensity-dependent through such loss mechanisms as multiphoton absorption and plasma generation. For the two pulses considered here, the long pulse is much lower in instantaneous intensity due to the facts that it is generated by a modulation of only a small fraction of the original bandwidth, and this much smaller quantity of energy is distributed over a much longer span of time than the original broad-bandwidth short pulse.

If the short pulse is intense enough to undergo nonlinear loss mechanisms while the long, less intense pulse only undergoes linear loss, the 
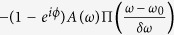
 term in the modulated pulse experiences less attenuation than the *A(ω*) part of the pulse. In the (not realizable in practice) limit of total attenuation of the short pulse and no attenuation of the long pulse, the observed intensity spectrum of the pulse will be proportional to |(1 − *e*^*iϕ*^)*A(ω*)|^2^ over the modulated bandwidth, which for the case of a phase flip (*ϕ* = *π*) is |2*A(ω*)|^2^, a factor of 4 greater than the pulse before the nonlinear propagation. In this manner, a time-domain attenuation process can cause intensity of the pulse to be concentrated at selected frequencies in the spectrum. This is illustrated in Fig. 1g,h, where a sharp spectral spike is generated by attenuating the short pulse components either completely (Fig. 1g) or to 65% of its original intensity (Fig. 1h). Notice that the intensity of the spectral spike is greater than the intensity of the same frequency components of the initial pulse.

## Results and Discussion

**Figure 2:**
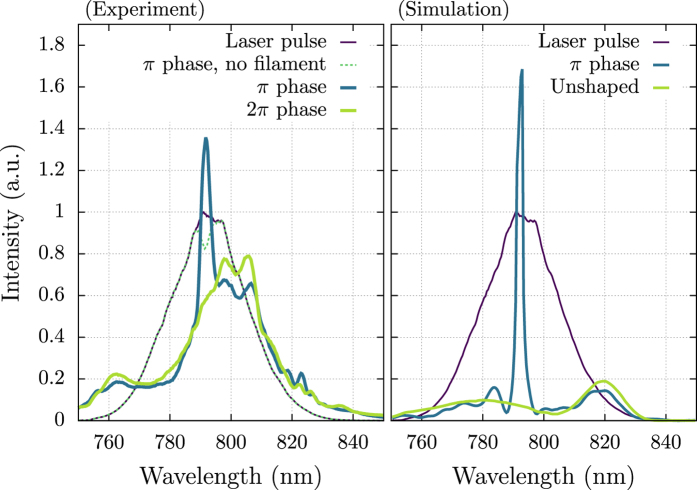
Resulting experimental and simulated spectra after filamentation when applying a *π* or 2*π* phase shift to a selected window of frequency components. Notice that applying a 2*π* phase shift removes the effect. All spectra were normalized by the peak of the laser pulse spectrum (no filamentation), which is shown for reference. The spectrum of the shaped laser pulse without filamentation is also shown for reference.

By applying a *π* phase shift (i.e. a phase flip) to a narrow spectral region (two pixels at the center of a spatial light modulator in a pulse shaper) of a 50 fs laser pulse (Coherent, Legend Elite), we observe a sharp peak arise in the emission spectrum of a femtosecond filament in the forward direction. The filament was approximately 7 cm long. This peak has a strong dependence upon the phase shift applied by the pulse shaper. As the phase applied is scanned from 0 to *π*, the peak rises to a maximum, and then disappears again as the phase is scanned further to a 2*π* phase shift. The measured spectra for *π* and 2*π* phase shifts are shown in Fig. 2. The spectra of the laser pulse and unshaped filament are given for reference. The spectrum of the shaped laser pulse without filamentation (acquired by removing the focusing lens) is also shown to demonstrate the importance of the self-guiding stage in this process. It is observed that the spike in the spectrum of the shaped filament has a larger spectral intensity than the same frequencies of the laser pulse without filamentation. The appearance of this intensity spike is also shown in the numerical simulation results of Fig. 2. The numerical results are integrated over the entire beam to account for the light collected by the integrating cavity. Some discrepancy in the behavior of the spectral wings between the experiment and numerical results is observed. This is possibly due to a combination of a number of effects such as the Raman effect^3^,^14^,^39^, ionization-induced effects^40^, or slight asymmetry in the experimental beam causing a portion of it to not undergo filamentation. Some discrepancy may also be due to diffraction effects from the spatial light modulator where the *π* phase step is applied within two pixels width (200 *μ*m). Evidence of this is shown in the spectrum of the shaped laser pulse without filamentation (Fig. 2, left). However, the qualitative agreement remains that when a *π* phase flip is applied to certain spectral ranges, the amplitude in those ranges after filamentation is enhanced.

**Figure 3:**
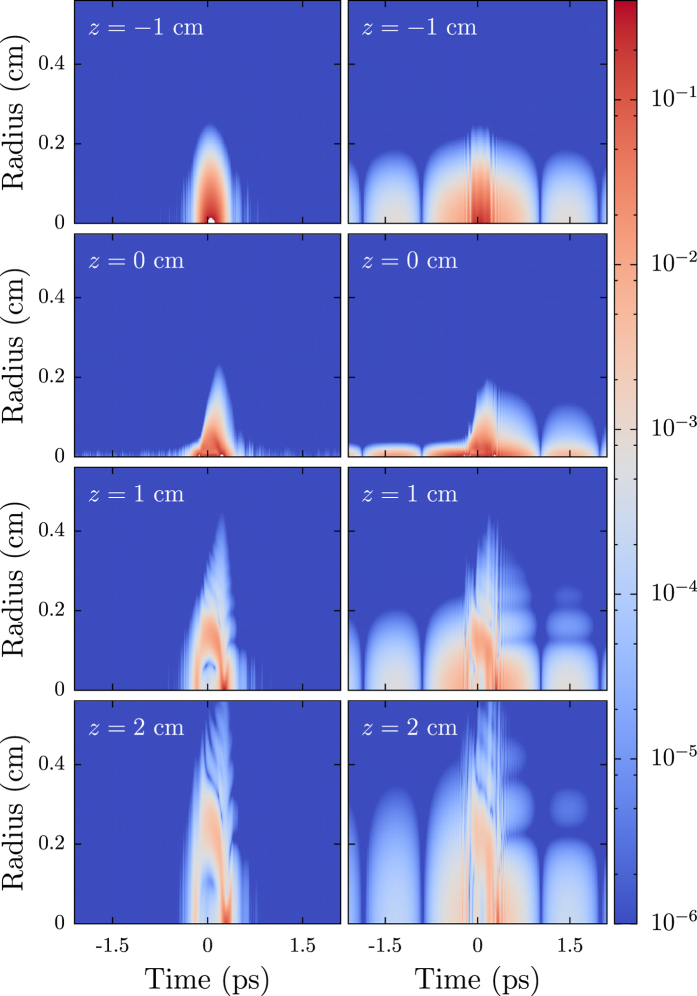
Snapshots of the intensity of the unshaped (left) and shaped (right) pulses as they propagate. The central component of both pulses undergoes filamentation, while the low-intensity leading and trailing edges do not. The trailing edge is also influenced by the plasma trail of the filament causing it to not focus as tightly as the leading edge. Linear focus is at *z* = 0 cm.

From the numerical results, insight to the dynamics of this effect are attained. In Fig. 3, numerical simulation results for shaped and unshaped pulses as a function of propagation position, radius from the center of the beam, and time are shown. Here we see that the propagation of the shorter, high intensity component of the pulse in the phase flipped case (right column) is essentially the same as the pulse in the unshaped case (left column). The difference arises from the longer, low intensity component of the pulse that does not undergo filamentation. Because the beam was focused, we see the leading edge of the pulse (negative time) undergo linear focusing and defocusing as it approaches and passes *z* = 0. The trailing edge (positive time) also focuses, but not nearly as much. Furthermore, ripples are observed in the trailing edge. This asymmetry between the behavior of the leading and trailing edges of the pulse is due to the plasma trail that is left in the wake of the filament.

**Figure 4:**
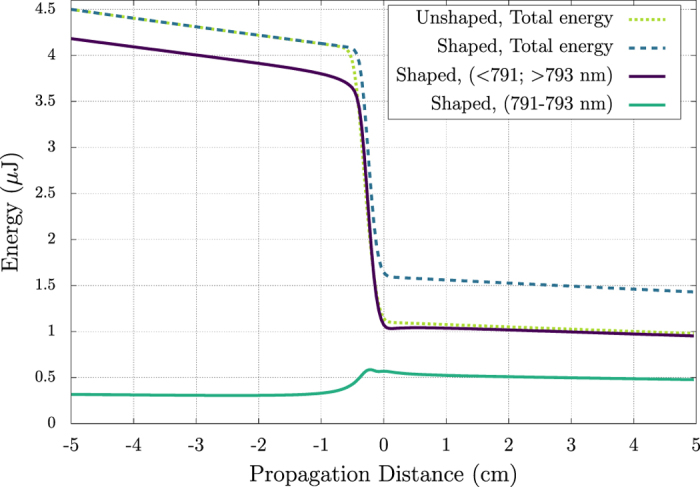
Energy of the simulated pulse propagation as a function of distance (linear focus is at 0 cm) for the case of a *π* phase flip at 791–793 nm (blue, dashed) compared to the case of no phase flip (light green, dashed). The total energy contained in the pulse drops as it propagates through filamentation. However, in the case of a *π* phase flip, this energy drop is considerably less. Plotting the energy contained in the spectral band of the phase flip (green, solid) versus the energy not in the phase flipped region (violet, solid), it is shown that energy is actually transferred to the frequencies that were phase flipped.

Integration of the spectrum of the various components of the pulse yields the energy contained in each component as a function of propagation distance. These are shown in Fig. 4, where the total energy in the shaped and unshaped pulse as a function of propagation are shown in green and violet, respectively. We see that the energy of the unshaped pulse decreases more than that of the shaped pulse as it undergoes filamentation. Also shown is the energy contained in the low-intensity and high-intensity components of the pulse for the phase flipped case. We see that the energy contained in the spectral region of the low-intensity component is actually increased as it undergoes filamentation, while the high intensity component is attenuated as usual.

**Figure 5:**
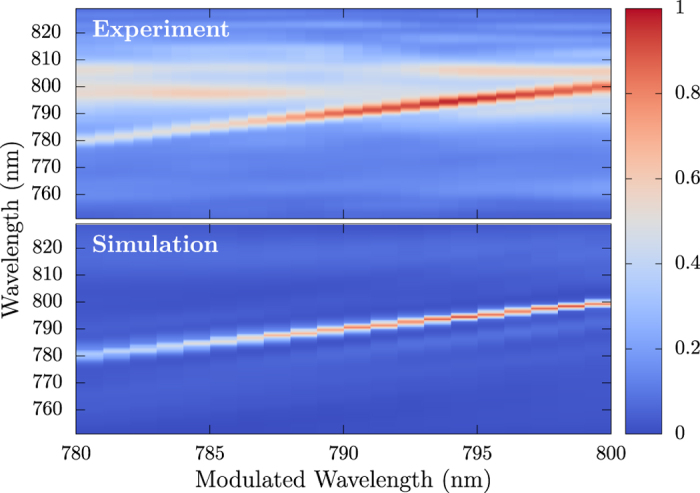
(Top) Spectra of the shaped filament as the modulated pixel is scanned across the spatial light modulator. The narrow peak created by phase modulation (see Fig. 2) follows as the pixel causing the phase shift is changed. (Bottom) Simulated spectra that show the same effect.

Furthermore, in simulation and experiment, the frequency of the peak generated by the amplitude concentration moves according to the frequency of light being modulated. This is achieved experimentally by simply scanning the modulating pixels across the spatial light modulator, and results are shown in Fig. 5. These results provide insights to applications of this technique. By selecting a pixel in a computer controlled spatial light modulator, we are able to tune the frequency of the narrow-bandwidth component of the pulse. Thus, a narrow-bandwidth laser pulse can be created tunable to any frequency within the limits of the original bandwidth of the femtosecond laser pulse.

**Figure 6:**
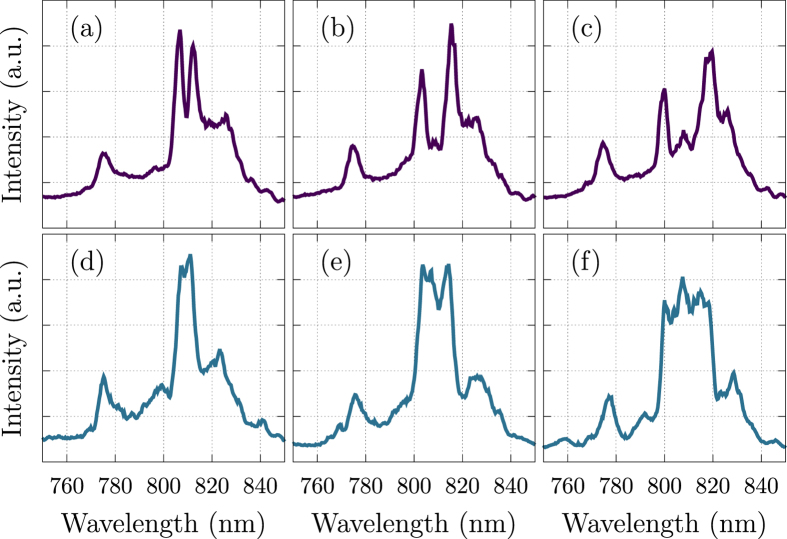
Spectra of Shaped Filament for different phase mask configurations. Here, two narrow intensity spikes (top row) are formed or one wide intensity spike (bottom row) is formed by correctly choosing which pixels of the spatial light modulator to modulate. The phase mask configurations used to obtain these spectral patterns are specified in the text. There is a possibility to create many different shapes in the output spectrum.

It is also possible to have multiple peaks as shown in the top row of Fig. 6. Here, a *π* phase shift is applied experimentally to two pixels with a gap between them in the spatial light modulator. In Fig. 6a, the two modulating pixels have a gap of six pixels with zero phase shift between them. In Fig. 6b, the gap is increased to 14 pixels, and 22 pixels in Fig. 6c. The peaks separate according to the modulating pixel separation. This demonstrates the ability to encode information onto the laser pulse in the form of simple phase flips, and the information is decoded using a simple spectrometer. Other spectral shapes can also be formed such as those shown in the bottom row of Fig. 6. Here, subfigures d,e, and f have the same configuration as a,b, and c except that instead of a gap of non-modulating pixels between the two modulating pixels, a set of 6,14, and 22 pixels respectively are all set to apply a *π* phase shift. Thus, to within the resolution limits of our spatial light modulator, we can control which frequencies to amplify.

## Conclusion

We have experimentally and theoretically demonstrated an example of amplitude concentration in a femtosecond filament. A *π* phase shift is applied to a laser pulse by a 4-f pulse shaper resulting in a pulse composed of a superposition of a long (narrow-bandwidth) pulse component with low intensity and a short (broad-bandwidth) pulse component with high intensity. Through intensity-dependent attenuation from femtosecond filamentation, energy is transferred into the spectral regions where the phase shift was applied. This amplitude accumulation process in femtosecond filaments is due to the nonlinear effects associated with filamentation; namely, nonlinear absorption due to ionization. It should be possible to generate similar results with any attenuating effect that has a similar threshold-like behavior, thereby attenuating the broad-bandwidth portion of the shaped pulse while leaving the narrow-bandwidth portion unaffected. Furthermore, the efficiency of this process may be improved by modifying the pulse characteristics (pulse duration, filament length, etc.) to increase the degree of nonlinear loss experienced by the high intensity component of the pulse.

Computer controlled spatial light modulators provide control over this result. Not only can the wavelength of the intensity spike be adjusted by choosing which pixel to modulate, but the height of the intensity spike can also be set by choosing the phase of the modulation applied. In this manner, it is possible to encode information into spectral phase jumps by the spatial light modulator. The information can then be decoded into measurable intensity spikes.

## Methods

### Experimental Setup

**Figure 7:**
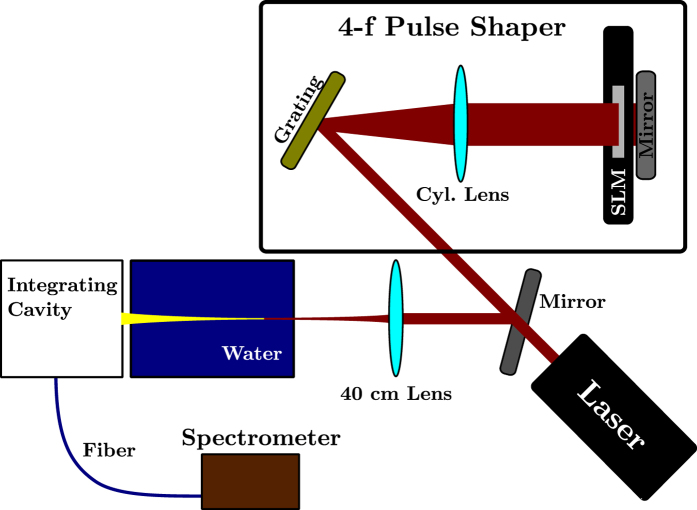
Experimental setup. A pulse shaper is used to apply a *π* phase shift to a narrow window of frequencies. This shaped pulse then undergoes filamentation in water. The resulting pulse has a modified spectral amplitude.

The experimental setup is depicted in Fig. 7. The phase of a 50 fs laser pulse from a Coherent Legend Elite system with a center wavelength near 800 nm is modulated by a 128 pixel Meadowlark liquid crystal spatial light modulator (D3128). This spatial light modulator is placed at the Fourier plane of a 4-f pulse shaper constructed using a 600 groove/mm holographic grating (Edmund Optics, NT47-555) and a cylindrical lens with a 20 cm focal length (Thorlabs, LJ1653L1). This pulse shaper was constructed in a folded geometry^31^ with a slight vertical separation between the output and input pulse. The pulse after the pulse shaper (8 *μ*J/pulse) is then focused into a 15 cm long tank of tap water using a 40 cm focal length lens (Thorlabs, LA1422). A filament is induced, and the light emitted in the forward direction is collected by an integrating cavity (home-built, 2.5 cm diameter entrance aperture) and measured by an Ocean Optics USB 2000+ spectrometer (1.3 nm resolution). The intensity of the spectra were calibrated using a Coherent PM10 power meter. Changing the phase mask applied by the spatial light modulator alters the resulting spectrum of the filament’s forward emission. Note that it is important to consider chromatic dispersion in water, since the group-velocity dispersion (GVD) coefficient is large for these wavelengths. However, in the regime of our pulse parameters (*t*_*p*_ = 50 fs, *w*_0_ ~ 100 *μ*m, *P*_peak_ ~ 30 *P*_cr_), chromatic dispersion cannot stop self-focusing and plasma generation^38^,^41^. Furthermore, in this regime, nonlinear absorption plays a key role in filamentation^38^.

In order to compare the initial spectrum of the laser pulse to the spectrum of the forward emission, measurements of both spectra are taken after the tank of water, thus accounting for linear absorption from the water. Filamentation is induced by the focusing from the 40 cm lens placed before the tank of water. Thus, by collecting the spectrum with and without this lens we are able to compare the emission spectrum of the shaped and unshaped filament to the laser pulse without filamentation.

However, because the laser is focused into the water in one case and not in the other, coupling into the spectrometer requires an integrating cavity to collect the light emitted into the forward direction (≤5° from optical axis). Thus, the spectral measurements are comparable regardless of the focusing geometry. The intensity measurement of the spectrometer is also further calibrated by normalization using a power measurement for each case.

Power loss due to reflection from the uncoated focusing lens also introduces inconsistency when comparing the intensity of the spectrum with the lens to the spectrum without it. We measured and accounted for this power loss in the normalization process of the data. Thus, we have obtained a calibrated measurement of spectral intensity for shaped and unshaped laser pulses with and without filamentation.

### Numerical Simulations

Our experimental results are compared to theory via numerical simulations where we use the generalized nonlinear Schrödinger equation for the field envelope *A* ≡ *A(t, r, z*), modified to include ionization effects^2^,^3^,^42^,^43^:


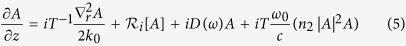


Here *r* is the radial coordinate, 
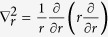
 is the transverse Laplacian, *z* is the propagation coordinate, 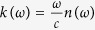, *ω* is the radiation frequency, *n(ω*) is the frequency dependent linear refractive index^44^, 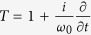 is the self-steepening operator, *t* is the retarded time, *ω*_0_ is the central frequency of the pulse, *n*_2_ = 2 × 10^−4^ cm^2^/TW^45^ is the nonlinear refractive index, and 
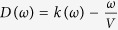
 is the dispersion operator, 
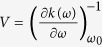
 being the group velocity.

Ionization effects are included through the 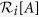 term:


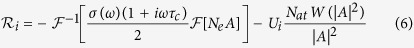


where 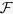 is the Fourier transform from *t* to *ω*, 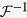 is the inverse Fourier transform, 
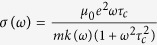
 is the inverse bremsstrahlung cross-section, *e* is the electron charge, *m* is the electron mass, *μ*_0_ is the vacuum permeability, *τ*_*c*_ is the electron momentum transfer time, *N*_*at*_ = 3.3 × 10^22^ cm^−3^ is the number density of water molecules, *W*(|*A*|^2^) is the single-molecule photoionization rate (calculated through Perelomov-Popov-Terent’ev formulas^46^,^47^), *U*_*i*_ = 6.5 eV is the effective ionization potential of water. The electron density *N*_*e*_ ≡ *N*_*e*_(*t, r, z*) is found from the equation


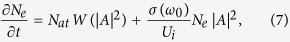


which accounts for photoionization and impact ionization. Simulations are started 5 cm before linear focus (*z* = −5 cm). The shape of the input pulse without a phase flip is calculated from experimental spectra, assuming the pulse is Fourier-limited at the cell entrance. While the experiment may deviate slightly from this assumption, the results should remain similar because of the robust nature of filamentation above threshold. The key point here is that the main pulse is well above threshold while the phase-shifted part of the pulse is well below threshold. These simulations have been performed in parallel codes using supercomputer complex of Lomonosov Moscow State University.

## Additional Information

**How to cite this article**: Thompson, J. V. *et al*. Amplitude concentration in a phase-modulated spectrum due to femtosecond filamentation. *Sci. Rep.* **7**, 43367; doi: 10.1038/srep43367 (2017).

**Publisher's note:** Springer Nature remains neutral with regard to jurisdictional claims in published maps and institutional affiliations.
